# Assembly of Copolymer and Metal−Organic Framework HKUST-1 to Form Cu_2−x_S/CNFs Intertwining Network for Efficient Electrocatalytic Hydrogen Evolution

**DOI:** 10.3390/nano11061505

**Published:** 2021-06-07

**Authors:** Yuanjuan Bai, Yanran Li, Gonggang Liu, Jinbo Hu

**Affiliations:** 1 Hunan Province Key Laboratory of Materials Surface & Interface Science and Technology, College of Materials Science and Engineering, Central South University of Forestry and Technology, Changsha 410004, China; liugonggang@csuft.edu.cn; 2 State Key Laboratory of Molecular Engineering of Polymers, Department of Macromolecular Science, Fudan University, Shanghai 200438, China; 17110440007@fudan.edu.cn

**Keywords:** assembly, metal-organic frameworks, hydrogen evolution reaction, Cu_2−x_S

## Abstract

The construction of complex intertwined networks that provide fast transport pathways for ions/electrons is very important for electrochemical systems such as water splitting, but a challenge. Herein, a three dimensional (3-D) intertwined network of Cu_2−x_S/CNFs (x = 0 or 0.04) has been synthesized through the morphology-preserved thermal transformation of the intertwined PEG-*b*-P4VP/ HKUST-1 hybrid networks. The strong interaction between PEG chains and Cu^2+^ is the key to the successful assembly of PEG-*b*-P4VP nanofibers and HKUST-1, which inhibits the HKUST-1 to form individual crystalline particles. The obtained Cu_2−x_S/CNFs composites possess several merits, such as highly exposed active sites, high-speed electronic transmission pathways, open pore structure, etc. Therefore, the 3-D intertwined hierarchical network of Cu_2−x_S/CNFs displays an excellent electrocatalytic activity for HER, with a low overpotential (η) of 276 mV to reach current densities of 10 mA cm^−2^, and a smaller Tafel slope of 59 mV dec^−1^ in alkaline solution.

## 1. Introduction

Electrochemical water splitting is a critical energy conversion process for producing clean and sustainable hydrogen, which is composed of two half-cell reactions: oxygen evolution reaction (OER) and hydrogen evolution reaction (HER) [1,2,3] Electrolysis is a process that consumes electricity, therefore a catalyst is needed to reduce the potential. Pt-based electrocatalysts exhibit the best performance for H_2_ evolution in strongly acidic electrolytes, however their HER activities are substantially diminished under alkaline conditions [4,5] Consequently, considerable attempts have been devoted to developing sustainable, highly efficient, and non-precious electrocatalysts to meet a target of Pt-based catalysts replacement. In recent years, transition metal sulfides (TMSs) have been widely investigated and have demonstrated their potential as HER catalysts due to their high catalytic activity and chemical stability [6] Metal-organic frameworks (MOFs) are an intriguing class of porous crystalline materials constructed by the coordination of metal ions or clusters with organic linkers [7,8,9,10,11] For example, HKUST-1({[Cu_3_(C_9_H_3_O_6_)_2_(H_2_O)_3_]}_n_, aka Cu-BTC) is one of the very first permanently porous MOFs, which has been widely studied for multiple applications [12,13,14] The microporosity and tunable functionality of MOFs make them ideal template precursors to fabricate various transition metal-based carbon composites, including TMSs, by means of the pyrolysis process under a specific atmosphere [6,15,16] However, MOFs may undergo structural collapse during pyrolysis, resulting in the dramatic decrease in surface area, the wreck of well-defined MOF pore/channel structures, and the uneven distribution of active components, which significantly reduces the electrochemical performances of the MOFs derived materials. Therefore, the performance of the existing MOF derivatives’ electrocatalysts is still struggles to match precious-metal-based materials.

One of the best ways to solve the above issues is the assembly of crystalline MOF nanoparticles into well-aligned one, two, or three dimensional (1-, 2- or 3-D) superstructures, used as an appropriate template as both scaffold and directing agent for the epitaxial growth of MOFs [17,18,19] After a pyrolysis process, the MOFs superstructures would be converted into transition-metal-based materials with ordered stacking and porous nanostructure. The choice of the template is vital to the success of the assembly. Commonly used templates include metal-based materials (e.g., Te nanowire [20]), carbon materials (carbon nanotubes (CNTs), carbon nanofiber (CNFs) and so on) and polymers [21,22,23] Among of them, 1-D polymer nanowires or nanofiber templates with unique high surface-to-volume ratio, adjustable size, and surface modifiability attract wide research interests [24,25] In particular, 1-D polyacrylonitrile (PAN) nanowires substrate prepared by electrospinning is the most widely used in many research studies [23,26] For instance, Centrone et al. reported a microwave irradiation approach to grow MIL-47 on 1-D PAN nanowires [27] Han et al. proposed that the uniform and stable growth of MOFs on PAN nanowires can be enhanced through two types of chemical modification methods [23] These studies have shown that the surface-exposed functional groups play a key role in the MOFs’ growth on the PAN substrate, which act as binding sites for metal species of the target MOFs. Nevertheless, due to the difficulties in controlling the number of functional groups, and the compatibility between functional groups and MOFs, MOFs may fall off and be unevenly distributed on the surface of PAN.

In 2012, Chen and coworkers reported 1-D poly (ethylene glycol)-*b*-poly(4-vinylpyridine) (PEG-*b*-P4VP) nanowires composed of a PEG shell and slightly crosslinked P4VP core [28] When the aspect ratio of nanowires is high enough, the 3-D intertwining network can be formed. The follow-up research confirmed that the PEG-*b*-P4VP intertwined nanowires are excellent templates for the nucleation and growth of small MOF crystals due to their high aspect ratio (an average diameter of 30 nm and length up to several microns) and original abundant nucleation sites for metal ions [29] In the electrochemical application, the construction of complex intertwined conductive networks is desired, which would provide fast transport pathways for mass and charge, and then enhance the performance of catalysts [30,31] It is likely that a TMSs/carbon composite intertwining network could be prepared by the vulcanization of the as-prepared PEG-*b*-P4VP@MOFs network; but this hypothesis remains unexplored.

Herein, we have successfully constructed a novel intertwined Cu_2−x_S/CNFs (x = 0 or 0.04; CNFs = carbon nanofibers) network. The self-assembling strategy is used to control the growth of HKUST-1 nanocrystals on the 1-D super long PEG-*b*-P4VP nanofibers to form PEG-*b*-P4VP@HKUST-1 composites under room temperature. The PEG shell of the PEG-*b*-P4VP nanofibers provide numerous nucleation sites for HKUST-1, while the P4VP core with a positive charge repels the metal ions (precursor of the HKUST-1). As a result, hybridization of the PEG-*b*-P4VP nanofibers by the HKUST-1 occurs selectively in the PEG shell. Afterwards, the Cu_2−x_S/CNFs composite materials with an intertwined network structure can be prepared with un-changed morphology by using in-situ thermal calcination PEG-*b*-P4VP@ HKUST-1 precursors. Compared with Cu_2−x_S/C composites derived from individual HKUST-1 crystal, Cu_2−x_S/CNFs significantly enhance the HER performance, which is strongly related to the novel network superstructure.

## 2. Results and Discussion

**Figure 1:**
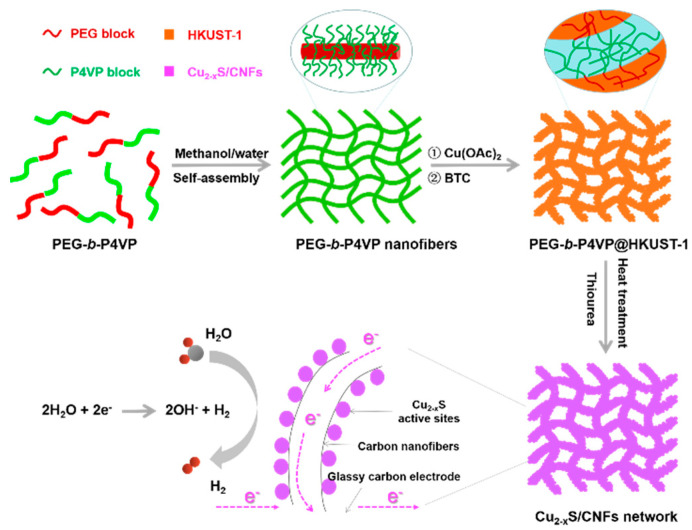
Schematic diagram of the synthesis of 3-D Cu_2−x_S/CNFs hierarchical network electrode.

Figure 1 shows the forming process of the 3-D network of Cu_2−x_S/CNFs nanocomposites. First, the core-crosslinked polymeric linear-like micelles with a PEG shell and a slightly crosslinked P4VP core, designated as PEG-*b*-P4VP nanofibers (NFs), is synthesized in a water/methanol mixed solvent according to a reported method [28]. Second, as the assembly substrate, PEG-*b*-P4VP NFs are dispersed in mixed solvent of water and N, N-Dimethylformamide (DMF) containing Cu(CH_3_COO)_2_·H_2_O (Cu(OAC)_2_). During this process, Cu^2+^ ions were favorably absorbed onto the PEG chain surface by the electrostatic interaction. Along to the previous step, trimesic acid (BTC) ligands solutions are added to assemble with Cu^2+^ ions via the coordination interactions [12,13,14]. Then, a uniform PEG/HKUST-1 hybrid shell is generated in-situ on the surface of the P4VP core, resulting in a PEG-*b*-P4VP@HKUST-1 composites with core-shell structure. Ultimately, the PEG-*b*-P4VP@HKUST-1 composites are converted into a 3-D hierarchical network of Cu_2−x_S/CNFs after the sulfurization reaction between PEG-*b*-P4VP@HKUST-1 and thiourea in an argon flow. The Cu_2−x_S/CNFs hierarchical network have many advantages for HER in alkaline solution including: (1) the continuous and conductive network of the CNFs with a hierarchically porous structure can enable fast charge and mass transfer; and (2) the high active specific surface areas of the Cu_2−x_S/CNFs offer abundant electrocatalytic active sites that are easily accessible in HER.

**Figure 2:**
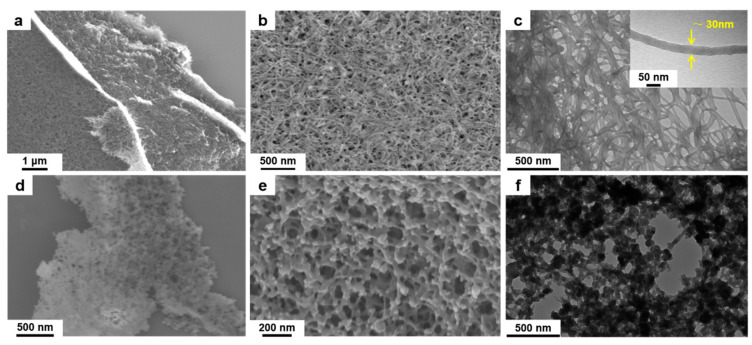
Morphology of the as-prepared (**a**–**c**) PEG-*b*-P4VP nanofibers and (**d**–**f**) PEG-*b*-P4VP@HKUST-1 hybrids as observed by SEM and TEM with different magnifications. The inset TEM image in Figure 2c shows the diameter of PEG-*b*-P4VP nanowire is approximately 30 nm.

**Figure 3:**
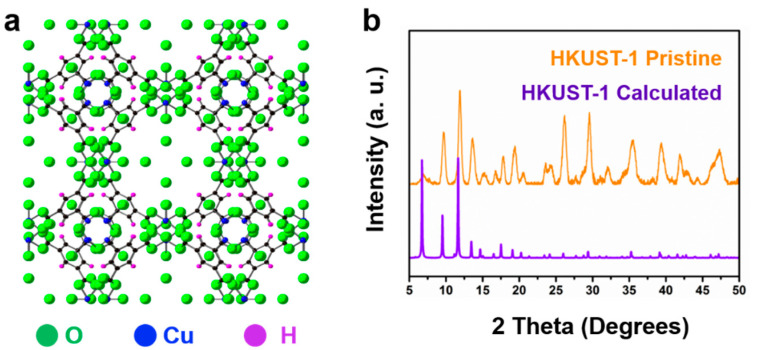
(**a**) The structure diagram of HKUST-1. (**b**) XRD pattern of the PEG-*b*-P4VP@HKUST-1 composites.

Field emission scanning electron microscope (FESEM) and transmission electron microscopy (TEM) in Figure 2a–c reveal that the PEG-*b*-P4VP nanofibers have a 3-D feature assembled by a series of stacked 1-D entangled ultrafine nanofibers with uniform diameters of about 30 nm and extra-long lengths above 2 μm. From the typical FESEM and TEM images in Figure 2d–f, we can see the structure of PEG-*b*-P4VP@HKUST-1 composites comprise a tightly crowded nanosized HKUST-1 crystal layer encapsulating the PEG-*b*-P4VP nanofibers with uniform size and well-defined shape. Notably, the generated composites inherit the intertwining network structure of their precursors. No unassembled irregular HKUST-1 crystals are observed, suggesting that the nucleation and growth of MOF crystals are localized on the surface of the PEG-*b*-P4VP templates. In addition, it is found that the polymer chains penetrate the crystalline structure of HKUST-1, and the particle size was less than 100 nm. Consequently, the interaction is PEG-*b*-P4VP nanofibers and HKUST-1 crystals, as it is extended from the core surface to the deep crystalline structure, which is important for the stability of HKUST-1 nanoparticles. The calculation result shows that the HKUST-1 content in PEG-b-P4VP/HKUST-1 composites is around 91 wt % (experimental section, Supporting Information). Because the individual composite nanofibers were dispersible in the suspension, the resultant architecture of the 3-D network shows a loose morphology (Figure S1). Figure 3 shows the X-ray diffraction (XRD) patterns of the PEG-b-P4VP/HKUST-1 composites. Based on the HKUST-1 structure data [12,13,14], the observed pattern and the simulated pattern show high similarity, confirming the formation of pure crystalline HKUST-1. It is found that the packing density of the HKUST-1 crystals be tuned by simply adjusting precursor concentration (Figure S2). In other words, the number of nucleation sites are controllable. As the addition of Cu^2+^ and organic ligands increased, the number of HKUST-1 nanoparticles on the surface of PEG-*b*-P4VP nanofibers gradually increased. In the end, the 0.24 g of Cu(OAC)_2_ and 0.21 g of BTC ligands were selected to apply for defined PEG-*b*-P4VP@HKUST-1.

**Figure 4:**
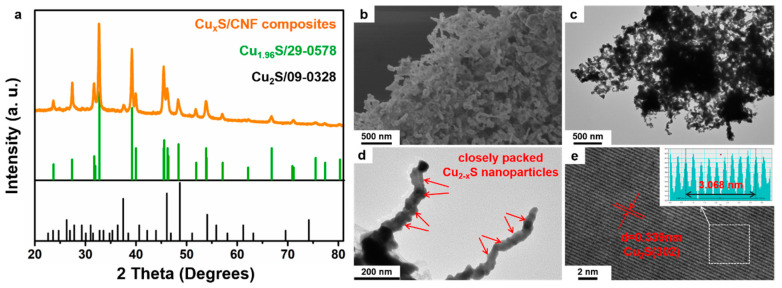
(**a**) XRD pattern, (**b**) SEM, (**c**,**d**) TEM and (**e**) HRTEM images of Cu_2−x_S/CNFs composites (inset image is the intensity plot of d-spacing for the (302) plane of Cu_2−x_S in e).

Characterization using XRD confirmed the structural identity and phase purity of the as-synthesized samples. As shown in Figure 4a, the XRD peaks of the final product after thermal treatment are well matched to those of the typical crystalline structures of Cu_2_S (JCPDS card no 09-0328) and Cu_1.96_S phase (JCPDS card no 29-0578). Therefore, the value of x can be determined to be 0 or 0.04. SEM (Figure 4b) and TEM (Figure 4c) images show that the 3-D intertwining network structure can be well-maintained for the Cu_2−x_S /CNFs even after calcination with the presence of thiourea under 400 °C. Closer observation of the Cu_2−x_S /CNFs (Figure 4d) reveals that Cu_2−x_S nanoparticle with coarse surfaces are uniformly packed along the ultra-long CNFs. No scattered Cu_2−x_S nanoparticles are observed, suggesting that the nanoparticles are sturdily attached to the CNFs. High-resolution TEM (HRTEM) (Figure 4e) observes clearly resolved and well-defined lattice fringes, revealing the high crystallinity in agreement with XRD results. The distance between the adjacent lattice planes is 0.339 nm, corresponding to standard spacing of (302) plane of Cu_2_S. By contrast, for individual HKUST-1 crystals prepared from the assembly of Cu(OAc)_2_ and BTC ligands without the PEG-*b*-P4VP template, the collapse of the porous structure and random aggregation of the nanoparticles occurred after sulfurization (Figure S3). The product of the individual HKUST-1 crystals after sulfurization treatment is denoted as Cu_2−x_S/C. The X-ray spectrometry (EDS) spectrum of the Cu_2−x_S/CNFs (Figure S4a) shows Cu and S signals attributable to Cu_2−x_S and C signals to CNFs. The corresponding EDS element mapping images (Figure S4b–e) demonstrate the homogeneous distribution of Cu, S, and C elements in the hierarchical nanostructure.

**Figure 5:**
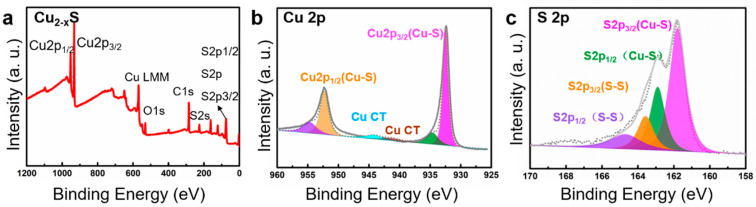
XPS survey spectra (**a**) of Cu_2−x_S/CNFs. High-resolution (**b**) Cu 2p and (**c**) S 2p XPS spectra for Cu_2−x_S/CNFs.

Electrochemical water splitting is a surface chemical process. The surface of materials plays a major role in determining catalysts behaviors. Consequently, the elemental compositions and valence states of Cu_2−x_S/CNFs network are studied in detail by X-ray photoelectron spectroscopies (XPS). Figure 5a displays clear a survey spectrum, which confirms the successful synthesis of Cu_2−x_S/CNFs. Regarding the Cu(2p) XPS spectrum as exhibited in Figure 5b, two strong peaks for Cu 2p orbit can be assigned to the Cu 2p_3/2_ and Cu 2p_1/2_ [32,33]. The binding energy of Cu 2p_3/2_ can be fitted into two peaks simulated at 932.7 and 934.6 eV [32,33]. The shakeup satellite peaks appearing at 943.7 eV might result from the partial surface oxidation of the samples when they made contact with air. In the S 2p region of the high resolution XPS spectra in Figure 5c, the binding energies (BEs) at 162.8 and 161.8 eV are ascribed to S 2p_3/2_ and S 2p_1/2_, respectively [34]. The two doublet peaks simulated at 163.7 and 164.9 eV correspond to the BEs of S-S bond. This result provided further evidence of the surface oxidation of Cu_2−x_S nanoparticles caused by air exposure. Raman spectroscopy in Figure S5 is used to investigate the nature of the carbon of Cu_2−x_S/CNFs. The two peaks located at 1351 and 1582 cm^−1^ match well with the D (disordered carbon) and G (graphitized carbon) bands of carbon, respectively. The D-band to G-band intensity ratio (I_D_/I_G_) of the samples is calculated to be around 1.07, implying the limited graphitization degree of Cu_2−x_S/CNFs.

**Figure 6:**
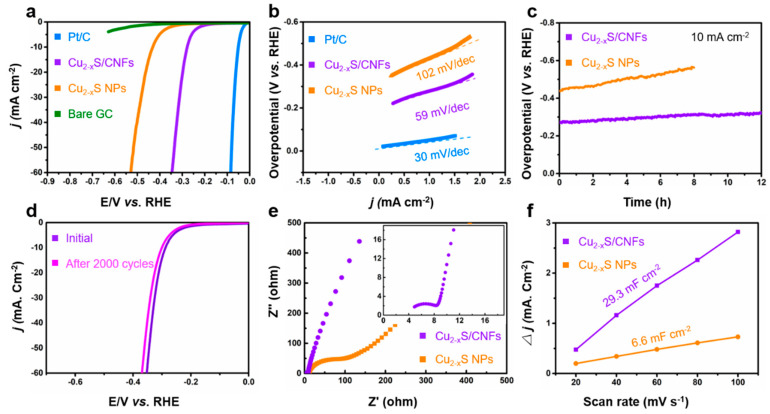
(**a**) LSV polarization curves and (**b**) corresponding Tafel plots of the Cu_2−x_S/CNFs, Cu_2−x_S/CNFs, 20% Pt/C, and a bare glassy carbon electrode derived from the polarization curves. (**c**) Chronopotentiometric curves at 10 mA cm^−2^ for Cu_2−x_S/CNFs and Cu_2−x_S/C composites over 10 h. (**d**) Accelerated HER polarization curves of Cu_2−x_S/CNFs. (**e**) Nyquist plots of Cu_2−x_S/CNFs and Cu_2−x_S/C composites at 300 mV overpotential in 1 M KOH (inset image is the magnified Nyquist plot of Cu_2−x_S/CNFs). (**f**) Capacitive current densities at 0.2 V vs. RHE as a function of scan rate of Cu_2−x_S/CNFs and Cu_2−x_S/C composites.

The electrocatalytic HER performance of Cu_2−x_S/CNFs is tested in 1.0 M KOH in a typical three-electrode setup with a scan rate of 5 mV s^−1^. Pt/C, bare glassy carbon (GC) electrode, and Cu_2−x_S/C composites are also examined for comparison. Figure 6a shows the linear sweep voltammetry (LSV) curves on the reversible hydrogen electrode (RHE) scale after iR correction. The bare GC electrode shows nearly no catalytic activity, while Pt/C exhibits excellent activity for HER. Cu_2−x_S/C composites show the limited HER performance with an overpotential of 432 mV at the current density of 10 mA cm^−2^. However, the as-prepared Cu_2−x_S/CNFs exhibit much superior HER activity compared to Cu_2−x_S/C composites, and only demand overpotentials of 276 and 337 mV to achieve current densities of 10, and 50 mA cm^−2^, respectively, fully demonstrating the importance of hierarchical intertwining network structures in the catalyst. The Tafel slope is an important indicator of electron-transfer kinetics and the rate-determining step in the HER process. The linear portions of the Tafel plots are fitted to the Tafel equation: η = b log j + a, where j is the current density and b is the Tafel slope. As shown in Figure 6b, Pt/C, Cu_2−x_S/C composites, and Cu_2−x_S/CNFs has Tafel slopes of 30, 102, and 59 mV dec^−1^, respectively. Based on the kinetic mechanism for alkaline HER, the HER over Cu_2−x_S/CNFs follows a Volmer−Heyrovsky mechanism, indicating that the Heyrovsky process (electrochemical desorption of hydrogen atoms) is the rate determining step [4,5,35] Electrochemical stability is another vital criterion to evaluate the new catalysts. As observed in Figure 6c, the Cu_2−x_S/CNFs retained 94% of its initial HER activity after a 12 h test, while the overpotential increase of the Cu_2−x_S/C electrode under the same condition was as high as 24% after only 8 h electrocatalysis. Meanwhile, the cyclic voltammetry (CV) durability tests of the Cu_2−x_S/CNFs electrode for HER in alkaline media is carried out at a scan rate of 100 mV s^−1^ (Figure 6d). After 2000 CV sweeps, the polarization curve shows a negligible difference compared with the initial curve, suggesting superior stability of Cu_2−x_S/CNFs in the long-term electrochemical process.

To further study the influence factors of catalytic kinetics, electrochemical impedance spectroscopy (EIS) and cyclic voltammetry (CV) are conducted. EIS analysis is performed to investigate the electrode/electrolyte interface properties of two catalysts with different structures. Figure 6e shows the Nyquist plots of the Cu_2−x_S/CNFs and Cu_2−x_S/C electrode at an overpotential of 300 mV (vs. RHE) in the frequency range of 100 kHz to 0.1 Hz. It can be observed that the charge-transfer resistance (R_ct_) is only 6.4 Ω for Cu_2−x_S/CNFs, but 58 Ω for the Cu_2−x_S/C electrode, in accordance with the HER results. This reflects the highly faradaic efficiency and fast electron transfer of the catalysts during the reaction. The smaller value of R_ct_ of Cu_2−x_S/CNFs electrocatalysts may be ascribed to their 3-D hierarchical intertwining network structure, which increased the contact of the active sites with the electrolyte, leading to a significant acceleration of the interfacial electrocatalytic reactions. We further tested the electrochemical double layer capacitances (C_dl_) (Figure 6f) of the catalysts by a simple cyclic voltammetry method, to relate the catalytic activity with the electrochemical surface area (ECSA) (Figure S6). The values are measured to be 29.3 and 6.6 mF cm^−2^, for Cu_2−x_S/CNFs and Cu_2−x_S/C, respectively, revealing the higher active surface area of Cu_2−x_S/CNFs. The large ECSA indicates that Cu_2−x_S/CNFs exposes higher accessible active sites, which is one of the possible reasons for the excellent HER performance.

## 3. Conclusions

In conclusion, Cu_2−x_S/CNFs with 3-D hierarchical intertwining network structures have successfully been designed and fabricated. They are derived from a 3-D network of PEG-*b*-P4VP@HKUST-1. The key to the successful assembly of the PEG-*b*-P4VP and HKUST-1 is the strong interaction between Cu^2+^ ions and PEG chains. Benefiting from the unique hierarchical structure and uniformly distributed active sites, the as-prepared Cu_2−x_S/CNFs Cu_2−x_S/CNFs exhibit high intrinsic HER activity in alkaline medium. The overpotential is 276 and 337 mV at the current of 10 and 50 mA cm^−2^, respectively. The Tafel slope is calculated to be 59 mV/decade, and the high activity can be maintained for more than 12 h. The assembly strategy of HKUST-1 and PEG-*b*-P4VP herein can be extended to the hybrid of other MOF-based materials and copolymer, which shows great potential, not only for catalysts, but also for gas sensors, energy storage, and environmental science.

## Data Availability

Data available in a publicly accessible repository.

## Supplementary Materials

The following are available online at https://www.mdpi.com/article/10.3390/nano11061505/s1, Figure S1: SEM and optical images of PEG-*b*-P4VP/HKUST-1; Figure S2: TEM images of PEG-*b*-P4VP@HKUST-1 composites at different weight of Cu(OAc)_2_ and BTC; Figure S3: SEM images of individual HKUST-1 crystals and Cu_2−x_S/C composites; Figure S4: EDS spectrum of the Cu_2−x_S/CNFs and the corresponding element mapping image; Figure S5: Raman spectrum of Cu_2−x_S/CNFs; Figure S6: CV curves of the Cu_2−x_S/CNFs and Cu_2−x_S/C composites with different scan rates.
