# Supplementary material for: Assembly of Copolymer and Metal−Organic Framework HKUST-1 to Form Cu2−xS/CNFs Intertwining Network for Efficient Electrocatalytic Hydrogen Evolution
Source: Nanomaterials (Basel). 2021 Jun 7;11(6):1505. doi: 10.3390/nano11061505 (PMC8227326; doi:10.3390/nano11061505)
Supplement: Supplementary file 1 [file nanomaterials-11-01505-s001.zip › nanomaterials-1239732-supplementary.pdf]

nanomaterials

MDPI

Supporting Information

Assembly of Copolymer and Metal–Organic Framework HKUST-1 to form $Cu_{2-x}S$/CNFs Intertwining Network for Efficient Electrocatalytic Hydrogen Evolution

Yuanjuan Bai [1,2,*], Yanran Li [2], Gonggang Liu [1] and Jinbo Hu [1,*]

1 Hunan Province Key Laboratory of Materials Surface & Interface Science and Technology, College of Materials Science and Engineering, Central South University of Forestry and Technology, Changsha 410004, China; liugonggang@csuft.edu.cn
2 State Key Laboratory of Molecular Engineering of Polymers, Department of Macromolecular Science, Fudan University, Shanghai 200438, China; 17110440007@fudan.edu.cn
* Correspondence: ; yuanjuanbai@126.com (Y.B.); hjb1999@hotmail.com (J.H.)

1. Experiment

1.1. Chemicals and Materials

All chemicals are of analytical grade and used without further purification. Cupric acetate monohydrate ($Cu(CH_3COO)_2{\cdot}H_2O$) (Cu $(OAC)_2$), Thiourea and trimesic acid (BTC) were purchased from Sigma-Aldrich (analytical grade). N, N-dimethylformamide (DMF) and ethanol absolute were purchased from Sinopharm Chemical Reagent. The water used in the experiment refers to ultrapure water.

1.2. Preparation of 3D PEG-b-P4VP@HKUST-1 Hybrids Network

First of all, $Cu(OAC)_2$ (0.24g) was completely dissolved in a mixed solvent of DMF (15mL) and H_2O (30mL) to form a transparent baby blue solution. The PEG-*b*-P4VP nanofibers stock suspension (2mg mL^{-1}, 4mL) was added into the above solution at room temperature for 30 min under magnetics stirring. Subsequently, the solution of BTC (0.21g) in a component solvent (DMF (15mL) and ethanol (30mL)) was rapidly poured into the above suspension and incubated at room temperature for 1h to complete the self-assembly. HKUST-1 were then grown on the surface of PEG-*b*-P4VP nanofibers template to form PEG-*b*-P4VP@HKUST-1 hybrids with a core-shell structure. The crude solid products were collected by centrifugation, followed by washing with ethanol twice and water once. The precipitate was then lyophilized and the obtained blue powder was denoted as PEG-*b*-P4VP@HKUST-1 hybrids.

The weight percentage (wt %) of HKUST-1 in the PEG-*b*-P4VP/HKUST-1 composites can be calculated as follows:

$$\text{wt \% (HKUST-1)}=[W(B)- W(C)]/ W(B) \times 100\%$$

Where W(B) and W (C) were the weight of PEG-b-P4VP/HKUST-1 and PEG-*b*-P4VP nanofibers composites, respectively. In the experiment, the weight of PEG-*b*-P4VP nanofibers is 8 mg (2mg mL^{-1}, 4mL). The weight of the final material of PEG-b-P4VP/HKUST-1 composites is ~ 90 mg by weighing with an analytical balance. The calculation results show that the wt % of HKUST-1 in the PEG-b-P4VP/HKUST-1 composites is around 91%.

1.3. Preparation of 3D Network of $Cu_{2-x}S$/CNFs Composite

The PEG-*b*-P4VP@HKUST-1 hybrids and Thiourea were placed at the center of a tube furnace and heated to 300 °C with a ramping rate of 5 °C min^{-1} under N_2. The samples were heated up to 400 °C at a heating rate of 1 °C min^{-1} and then held for 1h to fabricate the 3D network of $Cu_{2-x}S$/CNFs composites.

1.4. Characterization

Field-emission scanning electron microscopy (FESEM, Zeiss Ultra 55), the elements on the surface of sample were identified by energy dispersive X-ray spectroscopy (EDS). Transmission electron microscopy (TEM, FEI Tecnai G220 TWIN), and High Resolution TEM (HRTEM, JEM-2100F) were employed to observe the morphology and microstructure of the materials. Raman spectra were recorded by a Horiva (LabRam HR-800) spectrometer (532nm, 50 mW excitation laser). The powder X-ray diffraction (PXRD) pattern was collected using a PANalytical X'Pert PRO using Cu K_α radiation (40 kV, 30 mA) at the scan rate of 5° min^{-1} from 5° to 90° (2θ). X-ray photoelectron spectroscopy (XPS) spectra were recorded on a Thermo Scientific ESCALAB 250Xi with a monochromic Al K_α radiation (VG, USA) operated at 250 W. Raman spectroscopy was obtained from LabRAM HR Evolution.

1.5. Electrochemical Measurements

All of the electrochemical tests were carried out on an electrochemistry workstation (CHI660E, CH Instruments, Inc., Shanghai, China) by using a three-electrode mode in 1 M KOH aqueous solution. A glass carbon electrode, graphite bar, and Ag/AgCl electrode were used as working, counter, and reference electrodes, respectively. Typically, 5 mg of catalyst were dispersed in 1 mL of a mixture solution of 640 uL of water, 340 uL of ethanol and 20 uL of nafion solution, accompanied by ultrasonic dispersion for 1h to form a homogeneous slurry. Then, an aliquot of 7.5 μL was pipetted onto pre-polished glass carbon electrode and dried at room temperature (loading: 0.3 mg cm^{-2}). All the potentials in our paper were corrected to the reversible hydrogen electrode (RHE) using the equation E (RHE) = E (Ag/AgCl) + 0.059 pH+0.1976 V. Linear sweep voltammetry (LSV) profiles were recorded at a scan rate of 5 mV/s and double layer capacitances were derived from the slope of capacitive current versus scan rate plot. Cyclic voltammetry was applied to probe the electrochemical double layer capacitance at non-faradaic potentials (0.144 ~ 0.244 V *vs.* RHE) for estimating the effective electrochemical surface area (ECSA). All of potential in the LSV polarization curves were iR-corrected with respect to the ohmic resistance of the solution, unless specifically indicated.

2. Supporting Figures

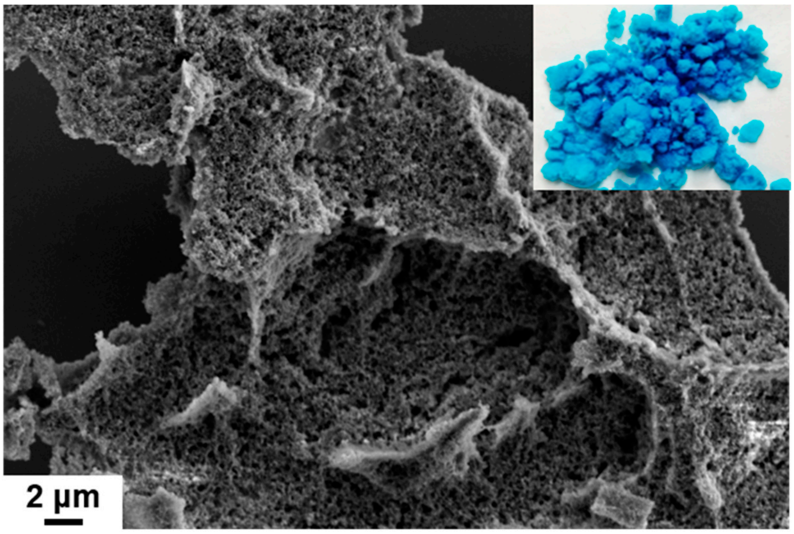

Figure S1. Low magnification SEM and optical images of the bulk-state PEG-b-P4VP/HKUST-1 composites network after drying. We can see the resultant architecture of the $Cu_{2-x}S$/CNFs network shows a loose morphology.

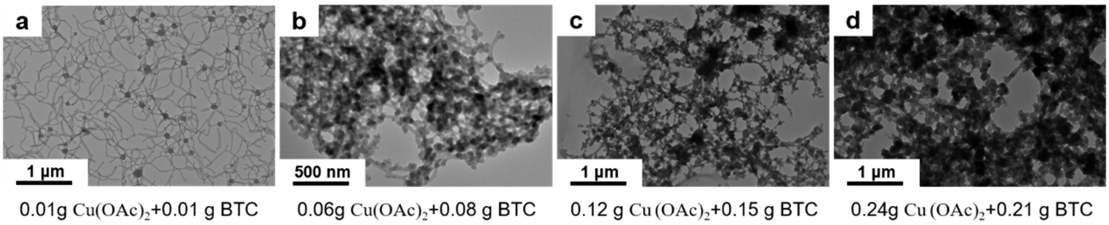

Figure S2. (**a**–**d**) TEM images of PEG-*b*-P4VP@HKUST-1 composites at different weight of $Cu(OAc)_2$ and BTC. As the addition of Cu^{2+} and organic ligands increased, the number of HKUST-1 nanoparticles on the surface of PEG-b-P4VP nanofibers gradually increased.

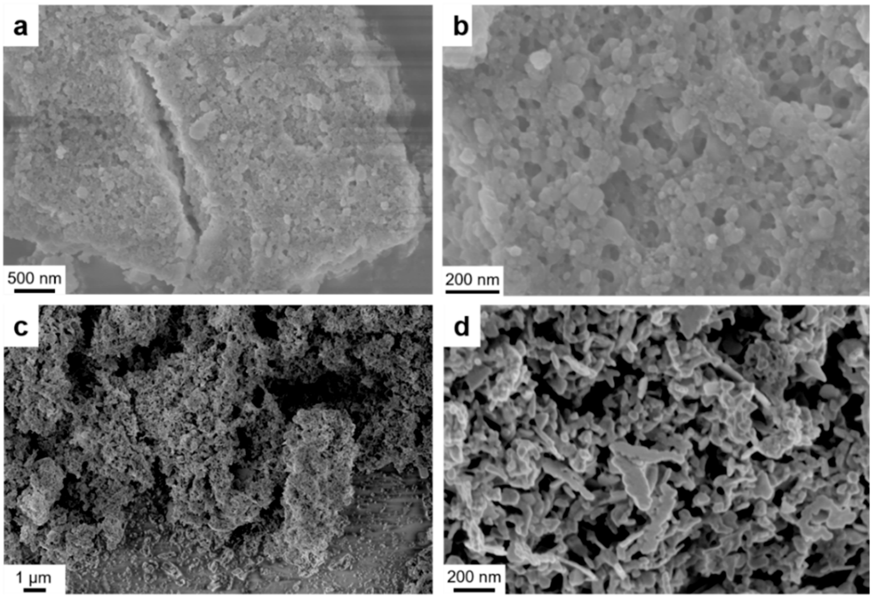

Figure S3. (**a**,**b**) Representative SEM images of individual HKUST-1 crystals prepared from the assembly of $Cu(OAc)_2$ and BTC ligands without the PEG-*b*-P4VP template. (**c**,**d**) SEM images for $Cu_{2-x}S$/C composites prepared from the sulfurization treatment of individual HKUST-1 crystals at 400 °C for 2h.

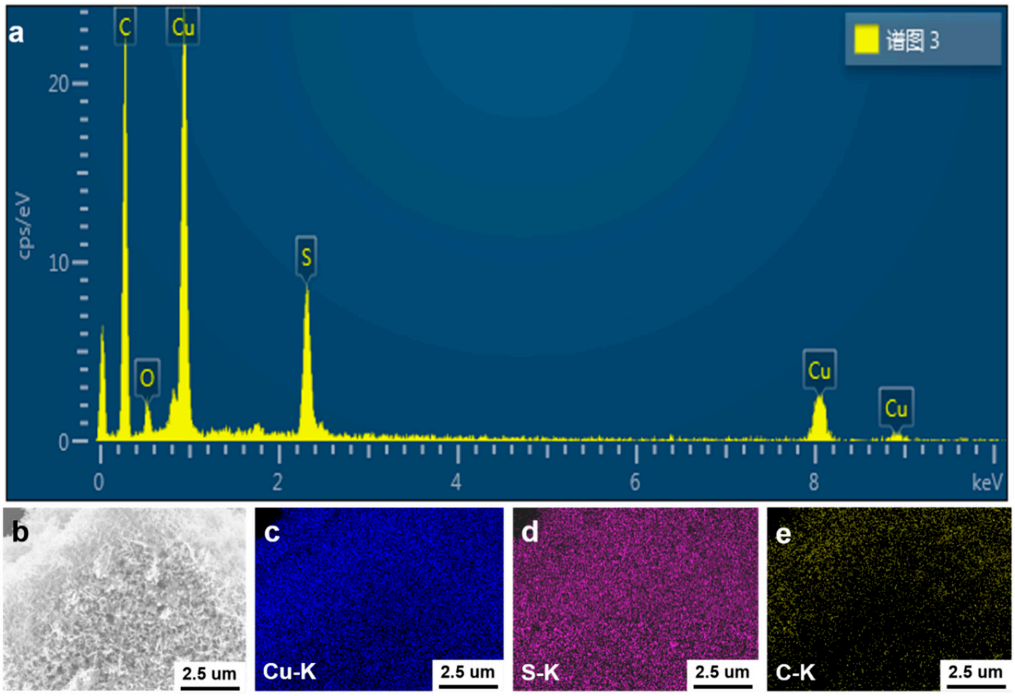

Figure S4. (**a**) EDS spectrum of the $Cu_{2-x}S$/CNFs. (**b–e**) element mapping of $Cu_{2-x}S$/CNFs showing the distribution of individual elements present in the composites.

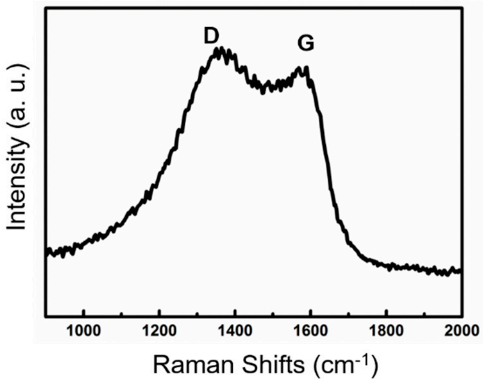

Figure S5. Raman spectrum of $Cu_{2-x}S$/CNFs network.

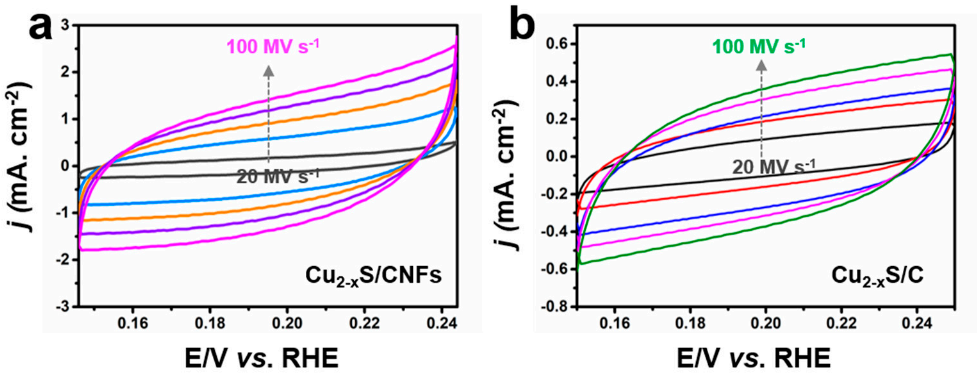

Figure S6. CV curves of the (**a**) $Cu_{2-x}S$/CNFs and (**b**) $Cu_{2-x}S$/C composites with different scan rates (20-100 mV s^{-1}) in the potential range 0.144–0.244 V vs RHE in 1 M KOH solution at room temperature.
